# A potential paradigm in CRISPR/Cas systems delivery: at the crossroad of microalgal gene editing and algal-mediated nanoparticles

**DOI:** 10.1186/s12951-023-02139-z

**Published:** 2023-10-10

**Authors:** Shuying Feng, Xin Xie, Junjie Liu, Aifang Li, Qianqian Wang, Dandan Guo, Shuxuan Li, Yalan Li, Zilong Wang, Tao Guo, Jin Zhou, Doris Ying Ying Tang, Pau Loke Show

**Affiliations:** 1 Medical College, Henan University of Chinese Medicine, Zhengzhou, 450046 Henan China; 2 Department of Pharmacy, Henan University of Chinese Medicine, Zhengzhou, 450046 Henan China; 3 Institute for Ocean Engineering, Shenzhen International Graduate School, Tsinghua University, Shenzhen, 518055 Guangdong China; 4 Department of Chemical and Environmental Engineering, Faculty of Science and Engineering, University of Nottingham Malaysia, 43500 Semenyih, Malaysia; 5 Department of Chemical Engineering, Khalifa University, P.O. Box 127788, Abu Dhabi, United Arab Emirates

**Keywords:** Algal-mediated nanoparticles, CRISPR/Cas system, Delivery system, Microalgae application, Gene editing

## Abstract

Microalgae as the photosynthetic organisms offer enormous promise in a variety of industries, such as the generation of high-value byproducts, biofuels, pharmaceuticals, environmental remediation, and others. With the rapid advancement of gene editing technology, CRISPR/Cas system has evolved into an effective tool that revolutionised the genetic engineering of microalgae due to its robustness, high target specificity, and programmability. However, due to the lack of robust delivery system, the efficacy of gene editing is significantly impaired, limiting its application in microalgae. Nanomaterials have become a potential delivery platform for CRISPR/Cas systems due to their advantages of precise targeting, high stability, safety, and improved immune system. Notably, algal-mediated nanoparticles (AMNPs), especially the microalgae-derived nanoparticles, are appealing as a sustainable delivery platform because of their biocompatibility and low toxicity in a homologous relationship. In addition, living microalgae demonstrated effective and regulated distribution into specified areas as the biohybrid microrobots. This review extensively summarised the uses of CRISPR/Cas systems in microalgae and the recent developments of nanoparticle-based CRISPR/Cas delivery systems. A systematic description of the properties and uses of AMNPs, microalgae-derived nanoparticles, and microalgae microrobots has also been discussed. Finally, this review highlights the challenges and future research directions for the development of gene-edited microalgae.

**Graphical Abstract:**

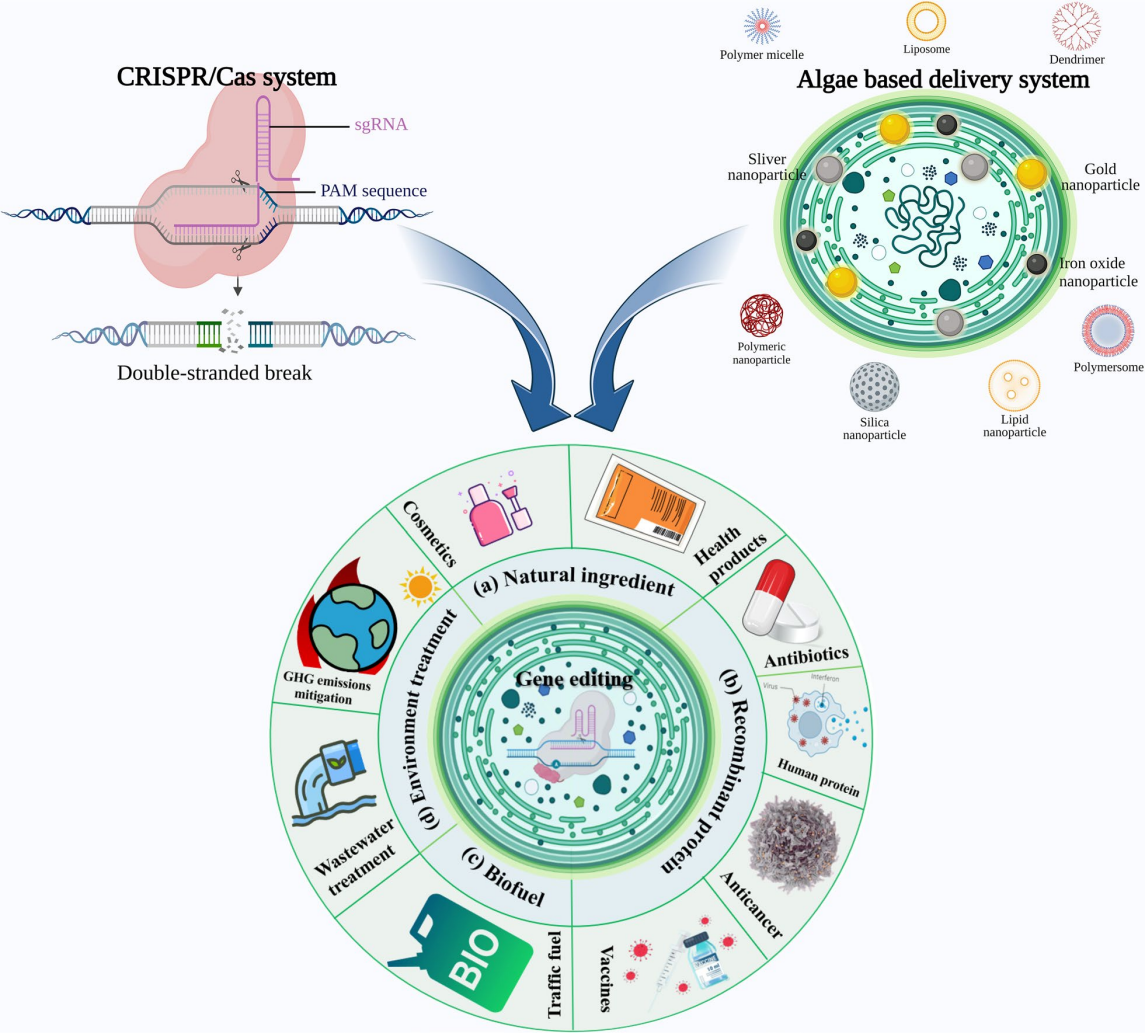

## Introduction

Following the urgent demand for energy and food, it is critical to discover an alternative source to alleviate these issues. Due to simple cultivation conditions, rapid growth rate and high photosynthetic efficiency, microalgae have emerged as the promising renewable energy resource. They also have a significant potential to construct a productive biorefinery which is the process of converting carbon dioxide into value-added compounds, for example proteins, vitamins, fatty acids, carotenoids, and nucleic acids. Microalgae are recognised as the third generation of biofuels with the potential for high-density cultivation system designs without compete with food or agricultural crops [1]. Additionally, microalgae are now capable of producing wide range of fuels, including biodiesel [2], hydrogen [3], syngas [4], etc. They may also serve as a raw material for functional food, natural dyes, and pharmaceutical drugs [5]. Studies on microalgae have primarily concentrated on developing integrative process and culture techniques, such as photobioreactor designs [6], harvesting approaches in downstream processes [7] and extraction techniques for high-value compounds [8]. However, this field still has some drawbacks, such as the low carbon fixation efficiency, low lipid accumulation rate and long cultivation period, which need to be overcome with more robust technology.

Many microalgal genomes have been sequenced to date, providing a definite genetic background and a compelling argument for genetic modification. Zinc finger nuclease (ZFN) [9], transcription activator-like effector-based nuclease (TALEN) [10] and the clustered regularly interspaced short palindromic repeats-associated system (CRISPR/Cas system) are examples of gene editing tools that offer an useful approach to deal with microalgal issues and achieve mass production. ZFN and TALEN are now severely constrained due to low editing efficiency, costly and laborious vector construction. The CRISPR/Cas system, in contrast, has become a reliable genome editing platform for gene correction, transcriptional regulation, disease modelling, and nucleic acids imaging. Its advantages include simplicity of target design, high editing efficiency, multiplex knock-in/out ability, low cost, and a quick cycle time [11]. Since then, CRISPR/Cas systems have been used as the treatment of infectious and metabolic diseases, creating sustainable techniques to produce chemicals and fuel as well as enhance the features of food crops. The disadvantages of CRISPR/Cas systems are off-target effects [12], variable efficiency, and inactive mutant [13, 14], despite the implementation of some strategies, for example rational design and modification of sgRNA, application of Cas variants, and improvement of the repair efficiency of HDR pathway.

An ideal delivery vector is essential for the efficient intracellular distribution of CRISPR components. Due to their superior biocompatibility in a homologous relationship, algal-mediated nanoparticles (AMNPs) have a tremendous potential to provide the CRISPR/Cas system for microalgal genetic editing. Furthermore, AMNPs provide a variety of properties, including anti-bacterial, anti-fungal, anti-cancer, anti-fouling, bioremediation, and biosensing activities [15]. This, green synthesis of AMNPs has drawn a lot of interest because it is safe, simple, sustainable, cost-effective, and eco-friendly. This review provides a thorough summary of the CRISPR/Cas system's delivery status using nanoparticles (NPs), with a focus on providing an in-depth description of AMNPs from the perspectives of their synthesis, features, classifications, and numerous applications. Some strategies, like as the use of a different Cas nuclease, better codon harmonisation, the addition of introns, and proper removal of the cell wall, have been suggested to tackle the limitations of CRISPR/Cas system in microalgae [16]. This review also examines the prospects of the AMNPs-delivered CRISPR/Cas system in microalgae, which offers a clear and crucial roadmap for advancing the use of gene editing in microalgae.

## Research status of microalgae gene editing

Although ZFN and TALEN have been applied in diatoms *Phaeodactylum tricornutum* and *Chlamydomonas reinhardtii* [17, 18], both technologies have not been widely used due to the limitations of laborious design steps, low editing efficiency and high off-target events. In comparison to ZFN and TALEN, the CRISPR/Cas system is regarded as a more sophisticated editing tool with several benefits, including the ease of design, increased effectiveness, and capacity to introduce mutations in many genes simultaneously [19]. In the past ten years, the CRISPR/Cas system has evolved from a groundbreaking genome-editing tool used in bacteria to a significant gene-editing tool utilised in plants, animals, and humans [20]. As a result, CRISPR technologies have advanced innovation by changing numerous eukaryotic genomes [21]. In the past, the CRISPR/Cas editing technology has been used to modify a number of microalgae species, including *Nannochloropsis oceanica* [22], *P. tricornutum* [23], *Thalassiosira pseudonana* [24], *Chlorella vulgaris* [25], and other microalgae species [26–40]. The editing process, transformation process, editing effectiveness, and experimental outcomes have all been explained in depth, as seen in Table 1 [41].

**Table 1:** Application status of CRISPR/Cas system in microalgae

Microalgae species	Microalgae subtypes	Editing method	Transformation method	Editing efficiency	Experimental results	References
*C. Reinhardtii*	*C. reinhardtii* CC-503	One plasmid-driven CRISPR/Cas9 system (*C. reinhardtii* codon-optimized Cas9)	Electroporation	46.7%	First successful transient expression of Cas9 and sgRNA in *C. reinhardtii*	[23]
	*C. reinhardtii* CC-124	CRISPR/Cas9 RNPs	Electroporation	0.17–40%	Mutagenesis of MAA7, CpSRP43 and ChlM gene	[24]
	*/*	CRISPR/Cas9 RNPs	Electroporation	0.56%	CpFTSY and ZEP gene knockout, increases photosynthetic productivity	[25]
	*C. reinhardtii* CC-400	Two plasmid-driven CRISPRi/dCas9-KRAB system	Glass beads	94%	Downregulating the expression level of CrPEPC1 gene to increase lipid synthesis	[26]
	*C. reinhardtii* CC-4349	CRISPR/Cas9 RNPs	Natural transformation	/	Knock-out the zeaxanthin epoxidase gene to stop the formation of lutein	[27]
	*/*	CRISPR/Cas12a RNPs	Electroporation	/	Exploring the mechanisms of single-strand templated DNA repair at CRISPR/Cas12a-induced DSBs in *C. reinhardtii*	[28]
	*C. Reinhardtii CC-4349, CC-124, and CC-503*	CRISPR/Cas9 RNPs	Electroporation	30%	Knock-in antibiotics resistance gene and YFP	[29]
*Synechococcus elongatus*	*Synechococcus elongatus* PCC 7942	One plasmid-driven CRISPRi/dCas9 system	Natural transformation	99%	The glgc gene was downregulated to increase succinate titer level	[30]
	*Synechococcus elongatus* UTEX 2973	Two plasmid-driven CRISPR/Cas9 system	Natural transformation	30–100%	Knock-out the nblA gene	[31]
*Synechococcus sp.*	*Synechococcus sp.* PCC 7002	Genomic integration CRISPRi/dCas9 system	Natural transformation	30–90%	Increasing central carbon flux by reducing carboxysome expression level	[32]
	*Synechococcus sp.* PCC7942	One plasmid-driven CRISPR/Cas9 system	Natural transformation	23–57%	Improvement of succinate synthesis using glgc gene knock-out and gltA/ppc gene knock-in	[33]
	*Synechocystis sp.* PCC 6803	One plasmid-driven CRISPRi/dCas9 system	Natural transformation	50–95%	Prevent the synthesis of carbon storage compounds	[34]
*N*. oceanica	*N*. oceanica CCMP1779	One plasmid-driven CRISPR/Cas9 system	Electroporation	45–90%	Generating non-transgenic marker-free nitrate reductase knock-out lines	[35]
	*N*. oceanica IMET1	CRISPR/Cas9 RNPs	Electroporation	93%	FnCas12a as the best performer for genome editing in *Nannochloropsis oceanica* IMET1	[36]
*Chlorella*	*/*	One plasmid-driven CRISPR/Cas9 system	Electroporation	67%	Enhancing lipid accumulation	[37]
	*C. vulgaris* UTEX395	CRISPR/Cas9 RNPs	Electroporation	/	Successful genome editing in *C. vulgaris* UTEX395 with CRISPR/Cas9 system	[38]
*T. pseudonana*	/	One plasmid-driven CRISPR/Cas9 system	Biolistic bombardment	61.5%	Two sgRNAs are used to induce a precise deletion in the urease gene of *T. pseudonana*	[39]
*P. tricornutum*	/	One plasmid-driven CRISPR/Cas9 system (*C. reinhardtii* codon-optimized Cas9)	Biolistic bombardment	25–63%	Mutagenesis of the CpSRP54 gene to increase the sensitivity to high intensity light	[40]

The problem of ineffective intracellular delivery with gene editing in microalgae persists despite varied success rates. An ideal transformation strategy and delivery vector are required to transport foreign DNA into cells. Among the transformation methods, such as electroporation [42], glass beads [43], particle bombardment [44], and *Agrobacterium tumefaciens*-mediated transformation [45], electroporation is the preferred method for microalgae transformation because it is rapid and highly effective in the generation of transformants (approximately 0.4 ~ 3 × 10^3^ transformants per µg of exogenous DNA) [42, 46]. There are few delivery carriers in CRISPR systems, such as viral vectors [47], extracellular vehicles [48], cell-penetrating peptides [49], and etc. AMNPs have a greater potential to deliver CRISPR/Cas components in microalgae gene editing due to better biocompatibility in a homologous relationship [50] than other delivery carriers in CRISPR systems. Their detailed delivery in microalgae have been described systematically as follows.

## NP-based delivery system for CRISPR/Cas components

### Characteristics of different delivery systems

Given the existing challenges of the CRISPR/Cas system, including as off-target effects [51], poor delivery efficiency [52], and unintended adverse effects [53], various techniques have been proposed to overcome these concerns [14, 51, 54]. Choosing the best delivery system has become the most important step, with strict requirements to boost not just loading efficiency, but also correct delivery to the specified location [55].

So far, several delivery mechanisms have sprung up, resulting in an exponential expansion in the distribution of CRISPR/Cas components (Table 2). Delivery mechanisms consists of three types which are viral vectors, non-viral vectors, and physical and chemical approaches [48, 56–60]. Viral vectors including adenovirus, adeno-associated virus and lentivirus [61–63], and physical methods like electroporation [64], microinjection [65], hydrodynamic injection [66] and ultrasound [67], are relatively well-established strategies for delivering CRISPR/Cas systems [68]. Using these two techniques, the delivery efficiency of CRISPR/Cas components was enhanced to around 98% [60, 69–71], signifying a substantial achievement in the field of gene editing. Viral vectors have become the most evident method for delivering genes owing to excellent transfer efficiency and stable gene expression [72, 73].

**Table 2:** Summary of common delivery strategies for CRISPR/Cas9 systems

Delivery systems	CRISPR/Cas9 formats	Delivery efficiency	Advantages	Disadvantages	Applications
Viral delivery					
AAV	pDNA	+ +	Low immunogenicity, non-pathogenic source, broad cell tropism	Limited packaging capacity, prolonged Cas9 expression, pre-existing neutralizing antibodies, high cost	In vivo
LV	pDNA	+ + +	Large packaging capacity, low immunogenicity, broad cell tropism	Potential for insertional mutagenesis, prolonged Cas9 expression	In vitro and ex vivo
AV	pDNA	+ +	Large packaging capacity, minimal genomic integration risk	High immunogenicity, difficult for large-scale production, pre-existing neutralizing antibodies	In vivo
Physical delivery					
Electroporation	pDNA, mRNA and RNP	+ + +	Suitable for multiple cell types, simple operation	Severe cell damage, nonspecific transfection	In vitro and ex vivo
Microinjection	pDNA, mRNA and RNP	+ + +	Highly specific and reproducible	Severe cell damage, low throughput, requirement of sophistication and manual skills	In vitro and ex vivo
Hydrodynamic injection	pDNA and RNP	+	Feasible for in vivo gene editing in small animals, highly efficient for liver transduction	Not suitable for large animals and clinical applications, non-specific and low efficiency, traumatic to tissues	In vivo
Non-viral delivery					
Lipid NPs	pDNA, mRNA and RNP	+	Good biocompatibility, minimal immunogenicity, feasible for large-scale production, temporal release of CRISPR-Cas9, all-in-one delivery, low toxicity	Lower delivery efficiency	In vitro and in vivo
Polymeric NPs	pDNA, mRNA and RNP	+	Minimal immunogenicity, feasible for large-scale production, relatively flexible functionalization, good pharmacokinetic control, large packaging capacity, all-in-one delivery	Lower delivery efficiency, variable biocompatibility and toxicity	In vitro and in vivo
Gold nanomaterials	pDNA and RNP	+ +	Higher delivery efficiency, good biocompatibility, unique optical properties and photothermal effect, relatively flexible functionalization	Potential toxicity in vivo at high concentrations	In vitro and in vivo
EVs	RNP	+	Excellent biocompatibility and negligible immunogenicity, high biostability relatively flexible functionalization, low toxicity	Tedious preparation process	In vitro and in vivo

+ denotes low; +  + denotes medium; +  +  + denotes high

Despite ongoing improvements in viral vectors [74], their applications in the delivery of CRISPR/Cas system are still hindered by the issues with immunogenicity [75], mutagenesis [76], limited packaging capacity as well as scale-up manufacturing [77, 78]. Combinatorial distribution of multiple components is a critical challenge that may prevent widespread and flexible implementation in the future [79]. Despite its great effectiveness in the laboratory, physical delivery was less practicable for in vivo distribution due to its low scalability [80, 81]. They are more suitable for in vitro applications than clinical translations because they can be conducted at the cellular level rather than the organism level [82]. Further, the implementation of physical delivery in non-specialist laboratories and high-throughput applications is difficult because to the expensive equipment and requirement of high skilled manipulation. Improper and unoptimized physical techniques may cause inevitable cell damage or even cell death [83, 84]. Alternately, it is desirable for the CRISPR/Cas system to deliver the product by changing it directly rather than causing alterations in target cells [80]. In view of this, chemical carriers provide a novel perspective due to their low immunogenicity, adjustable cargo size, and mass-production feasibility [85]. More importantly, the ease of alteration of chemical carriers may answer future issues in terms of targeting, biosafety, loading capacity, and spatiotemporal controllability, which are desirable options for in vivo precise gene editing delivery [86, 87]. Among the many chemical vectors, nanomaterial is an efficient platform to deliver small molecule medications, genes, peptides, and diagnostic agents [88, 89]. CRISPR/Cas components could be supplied via designed NPs with advantages such as precise targeting [90], high stability [91], safety [68], enhanced immune escape [92], and combinatorial delivery of several components [60].

### NPs delivery mechanism

The journey of NPs in vivo is difficult and accompanied with numerous obstacles, including disturbance of ‘protein corona’ [93], clearance by reticuloendothelial system, as well as the barriers of vasculature, extracellular matrix and endosome. In particular, the intravenous injection of NPs would face all of the difficulties of beginning from scratch, with the most challenging obstacle being the efficient access to organelles at the subcellular level [94]. The most important process for almost all successful NP-based carriers is endocytosis, which is mediated by target cells [95]. By controlling and mediating numerous signal pathways, NPs-based carriers were engulfed into endocytic vesicles through the invasion of plasma membrane [96–98].

**Fig. 1:**
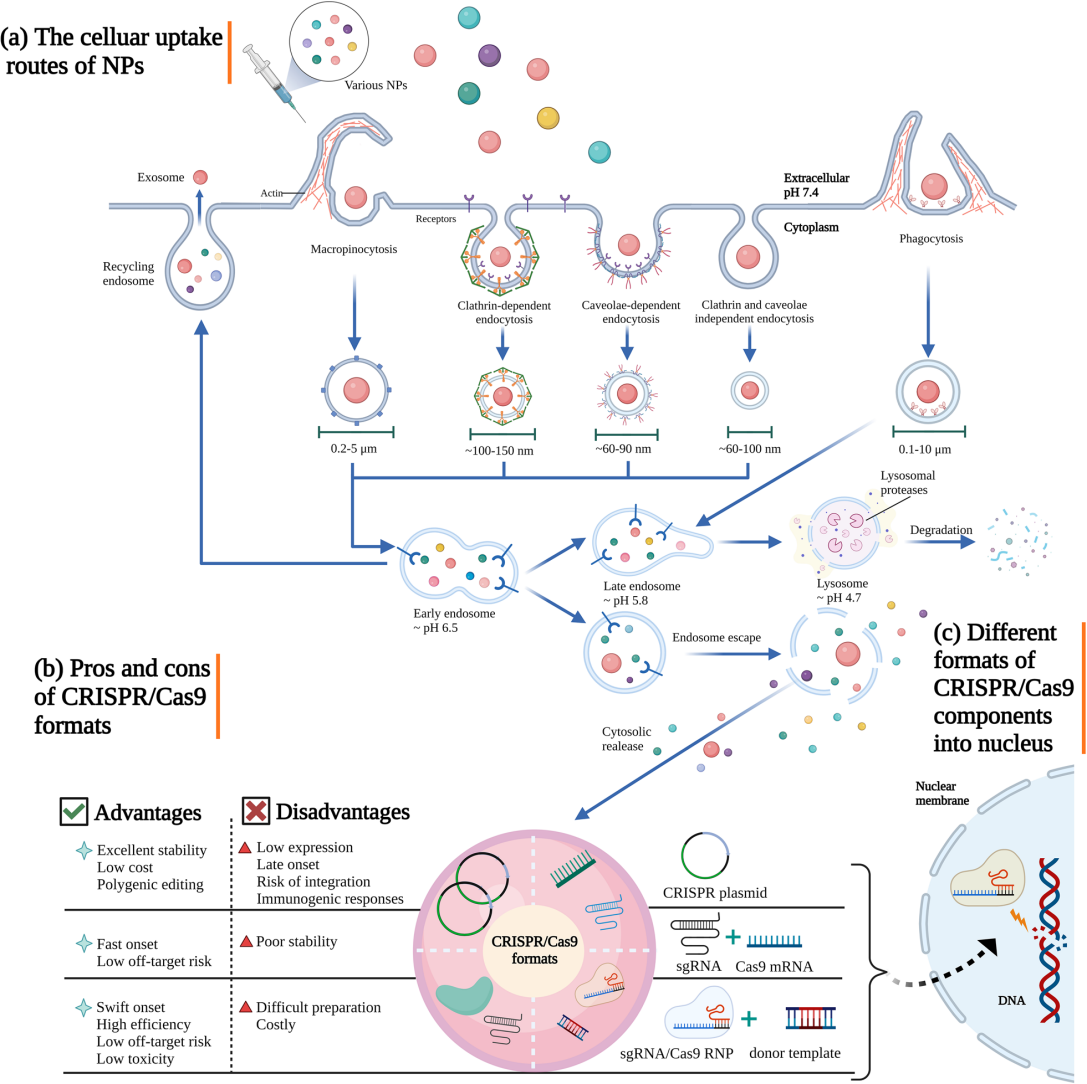
Delivery mechanism of NPs into cells. **a** The cellular uptake routes of NPs. **b** Pros and cons of CRISPR/Cas9 formats. **c** Different formats of CRISPR/Cas9 components into nucleus

Theoretically, these pathways are subdivided into five categories based on the specific lipid and transport protein types they contain, such as surface receptors, membrane lipids, and adaptor proteins. Phagocytosis, caveolin-dependent endocytosis, clathrin- and caveolin-independent endocytosis, and macropinocytosis are all types of endocytosis [99, 100] (Fig. 1a). The fate of NPs within the cells can be determined by many types of endocytosis, which depends on the factors including cell type, size, charge, and stiffness of NPs, as well as receptor interaction [101]. Among these mechanisms, the majority of receptor-bound NPs could be translocated into the cells by clathrin-mediated and caveolin-mediated endocytosis, while non-targeting NPs in small or large size will be taken up non-specifically by macropinocytosis or phagocytosis [102]. However, a certain lipid composition (mostly cholesterol) was required for clathrin- and caveolin-independent endocytosis, which was thought to be a direct entrance route [101]. However, the uptake mechanism of each item is distinct and has not yet been thoroughly investigated. When NPs become entrapped in an endosome during endocytosis, they are unable to perform their intended biological and therapeutic roles due to a lack of quick access to cytoplasm or cellular organelles. Endosomes were frequently acidified during ageing processes, with pH variations ranging from pH 6.5–6.8 to pH 5.2–6.0. Eventually, the acidic pH and enzymatic breakdown caused the cargoes to be destroyed [103]. As a result, the inability to exert biological effects is currently a key hindrance to nanomedicine efforts, necessitating the urgent development of endosome escape to increase the effectiveness of NPs delivery.

When NPs are subjected to the harsh environment of lysosome, endosome escape is a critical step in retaining the integrity of their cargoes and exerting their effects. According to the data, the greatest distribution effectiveness of NPs made of polymers and lipids was just 1–2%, and that the remaining 98% of NPs were useless because they were trapped in endosomes [104] or recycled back to the extracellular space [105]. It is frequently regarded as the rate-limiting step in the intracellular delivery of NPs-based systems [106, 107]. Therefore, an endosome escape technique could be used to transfer CRISPR/Cas components effectively without degrading them. However, various contentious ideas have been offered as endosome escape routes, such as proton sponge effect [108], lipid fusion with endosomal membrane [109, 110], nanoparticle swelling [111], membrane destabilisation [112], and cationic lipids induced hexagonal H_II_ conformation [113]. These mechanisms can be categorised into three types: membrane disruption [114], membrane fusion [115], and surface modification [116]. However, it is well known that all mechanisms with endosomal pH variation will be a strong indicator of endosomal escape, which is advantageous for the development of high-efficiency gene editing. Thus, investigating pH-responsive nanomaterials, such as NPs expansion [111, 117], polymer depolymerization [118, 119], and pH buffering materials [120, 121], could be a future study topic. In addition to endocytosis, NPs can be directly internalized into cytoplasm by passive membrane fusion or pore formation, which bypasses the endosome engulfment [110, 122]. However, the understanding of the trafficking of NPs is still evolving, thus additional efforts need to be done to confirm the precise mechanism. Developing novel methods and tools to dynamically observe membrane perturbation events and real-time cargo releases through visualisation of intracellular trafficking will be crucial for emerging biomedical applications to gain an in-depth understanding of cellular uptake process. Aside from the direct progressive approaches, much effort has been invested on the use of endocytosis inhibitors to promote and encourage advancement in the field.

### Selection of CRISPR/Cas formats

The ideal CRISPR/Cas format is essential for meeting the requirements for various applications, including plasmid DNA (pDNA), mRNA, and Cas9/sgRNA ribonucleoproteins (RNPs), to achieve high-efficiency gene editing [123]. When viewed in the context of NPs formulations and clinical or research application, each format offers unique characteristics [124, 125] (Fig. 1b). Due to its outstanding stability, affordable, and ease of preparation, CRISPR/Cas pDNA has become the most popular format. Additionally, it is practical to perform polygenic editing simultaneously at several sites using different sgRNA designs. However, the drawbacks of pDNA limited the applicability of the editing system. These drawbacks included the relatively low expression, delayed responsiveness, induction of immunogenic responses, and difficulty of encapsulating large molecules. Even worse, because Cas9 proteins are expressed for a longer period of time, on-target effects would be compromised and there would be a higher chance of uncontrolled plasmid sequence integration [126–129]. The delivery of mRNA or RNP complex demonstrated a rapid onset of gene editing in comparison to the pDNA format, bypassing the restrictions of pDNA and achieving functional complementation. The mRNA format can prevent undesirable insertional mutagenesis and makes it simpler to generate transient expression in the cytoplasm, which lowers the off-target effects [130–132]. However, Cas9 mRNA distribution is difficult because of the low stability which results from its fragile single-stranded structure and is susceptible to in vivo RNAse degradation [133]. Thus, attempts should be taken to improve its stability using different vectors or chemical modifications.

RNP complexes are the simplest delivery format, and they have the least amount of intracellular processing that can evade the transcription and translation process (Fig. 1c). To accomplish this, they equip components with a rapid onset and short duration, which results in substantially fewer potential side effects and cellular toxicity as well as high gene editing efficiency [134, 135]. Nevertheless, as a specific obstacle of RNP, exogenous risk and big molecule size would cause immunogenicity and difficulty in efficient nuclear entry, limiting the broad uses of the CRISPR/Cas system [136]. Additionally, the preparation of Cas9 proteins is time-consuming, complicated, and costly [137]. The compatibility between the cargo and the carrier is also important to consider, along with the reasonable choice of cargoes. Designing NPs should not compromise other qualities, such as loaded-cargo protection, targeting, and effective transfection, in order to accommodate a specific CRISPR/Cas system format [81]. In conclusion, every delivery metod hin a variety of formats has disadvantages of its own. Depending on the compatibility of the cargo and the carrier, as well as the requirements for diverse purposes, selecting the best CRISPR/Cas format is therefore essential for effective editing.

### NPs-based CRISPR/Cas delivery systems

The development of delivery carriers is currently trailing behind with regard to the distribution of CRISPR/Cas systems. Hence, there is an urgent need for sophisticated delivery systems with high efficiency, targeting, controllability, and safety characteristics. Synthetic nano-delivery systems, for instance lipid NPs (LNPs) [97], polymeric NPs (PNPs) [138], and gold NPs (AuNPs) [139], showed significant potential in enhancing the editing efficacy for CRISPR/Cas systems. These systems have the advantages of transient expression patterns, feasibility for mass-production at lower cost, all-in-one delivery, and low immunogenicity risk. These systems are also more flexible to deliver various cargoes for different purposes, encompassing RNPs, pDNA, mRNA, and donor DNA [140, 141]. In conclusion, nanotechnology holds the promise of overcoming the drawbacks of traditional delivery systems through cell-specific targeting, precise molecular transport to certain organelles, and other innovative strategies.

#### Lipid NPs

LNPs have been extensively investigated as the cutting-edge delivery platform for the drugs [142], CRISPR/Cas components [143], and vaccines [144]. They normally consist of four main components, i.e., key cationic or ionizable lipids complexed with negatively charged genetic material, phospholipids for particle structure, cholesterol for stability and membrane fusion, and PEGylated lipids to increase stability and circulation [145, 146]. The structure and formulation of LNPs protect the cargoes from enzymatic degradation and immunological responses, which subsequently facilitated their transportation into host cells for genome editing [147]. Ionizable lipids eventually replace permanently charged lipids due to their greater transfection efficiency and reduced cytotoxicity. Uncharged ionizable lipids are likely to adhere to the cell surface when the pH is neutral due to hydrophobic interactions or receptor-mediated endocytosis [148]. Ionizable lipids change to cationic form at lower pH to enable endosome escape and promote cargo release [113]. LNPs also have various advantages such as large cargo packaging capacity, good biocompatibility and bioavailability, low cell toxicity and immunogenicity, and mature industrial manufacturing technology. However, due to low drug loading and non-targeted biodistribution, it is challenging to achieve safe, efficient, and targeted administration of CRISPR/Cas components in vivo using LNPs [149]. The rational design or modulation of LNP formulations with the aim of obtaining optimum safety profile and efficient nucleic acid delivery would be a feasible technique to achieve the desired effects mentioned above [150, 151].

Developing new LNPs composition, such as NTLA-2001 LNPs [152] and 306-O12B-LNPs [153], can achieve targeting delivery of the CRISPR/Cas system with excellent editing efficiency and safety. In the case of treating transthyretin amyloidosis, the use of NTLA-2001 LNPs, an intravenous formulation made using a patented ionizable lipid, has demonstrated liver-targeted delivery. These LNPs display a consistent editing effect that is dosage dependent, safe, and long lasting, with maximum suppression seen after 12 months. The editing efficacy of transthyretin reached up to 93.7%, resulting in 87% decrease in protein concentration. In comparison to the gold standard LNPs (MC-3 LNPs, 14.6%), 306-O12B LNPs had a more effective liver-targeted delivery efficacy of around 38.5% [154], which offered the possibility of liver-specific administration in therapeutic applications. The high liver-targeted delivery efficiency could be attributed to the strong liver tropism caused by the active-targeting mechanism mediated by apolipoprotein E (ApoE) [113, 155]. Plasma ApoE attaches to the surface of LNPs and then interacts with the low-density lipoprotein receptor to achieve active endocytosis via ligand-receptor transport. Given this, modifying LNPs with appropriate ligands or targeting moieties may be an effective method for delivering drugs to specific organs [139, 141]. Similar to this, adding functional components to LNPs can reduce the risks associated with immunogenicity. TCL053-LNPs might preferentially transfer Cas9 mRNA and sgRNA into skeletal muscle due to the pH-dependent ionizable lipid TCL053. This approach could treat skeletal muscle disorders by giving repeated intramuscular injections because of its low immunogenicity. Nevertheless, each TCL053-LNPs injection could result in a long-lasting restoration of the dystrophin protein for at least a year [156].

Additionally, altering the proportion of LNPs formulation can achieve targeted and secure delivery for the CRISPR/Cas system. Multiple CRISPR cargoes could be selectively delivered to the targeted organs using a new technique called selective organ targeting (SORT), which involves changing the biodistribution of SORT LNPs [157]. The delivery to hepatocytes attained the highest specific transfection rate of 93% by including a supplementary component to the initial LNPs formulation. By constantly controlling the SORT molecule proportion, this system offered a reliable and designable platform for extrahepatic delivery, such as lung and spleen, which is also applicable to different NPs systems for targeted delivery. The proportion of various lipids in LNPs was then optimised using SORT and MC-3 LNP nanotechnology to create a lung-targeted LNPs-mediated CRISPR/Cas13d delivery system [158]. The data showed that, when compared to control groups, this system significantly decreased mRNA and protein levels of cathepsin L only in mouse lungs and reduced lung virus infectivity by two orders of magnitude, indicating an excellent strategy with exceptional efficacy, specificity, and safety. Since it is impossible to completely eliminate off-target events, efforts must be taken to address safety issues with the CRISPR/Cas system in order to build a perfect genome editing tool [60]. These efforts include sgRNA preoptimization, transient expression of the Cas9 protein, and targeted delivery to the intended tissue.

#### Polymeric NPs

Using multivalent charge interactions, PNPs condensed charged genome editing cargoes into nano-sized packages, shielding them from deactivation and promoting intracellular transit for genome editing [159, 160]. Different PNPs such as polyethylenimine (PEI), poly(lactic-co-glycolic-acid) (PLGA), poly(β-amino ester) (PBAE), poly(ethylene–glycol) (PEG), and amine-terminated polyamidoamine dendrimers have become very popular to deliver CRISPR/Cas components due to their excellent pharmacokinetic control, high cargo encapsulation, minimal immunogenicity, relatively flexible functionalization, and high bioavailability [78, 161]. More significantly, PNPs were able to accurately adjust the loading effectiveness and release kinetics to perform the editing for various objectives by varying their composition, stability, responsiveness, and surface charge characteristics [162]. Increasing the effectiveness of gene editing has increasingly become the significant route for the CRISPR/Cas system. PEI-based formulations, including branching PEI 25 kDa, are frequently utilised as the carriers for efficient delivery of CRISPR/Cas plasmids into the appropriate target cells when using PNP alone. In this technique, guide RNA and Cas9 are both expressed simultaneously, producing indel efficiencies (24.4%) that are comparable to Lipofectamine 2000 (27.9%) [163]. In contrast, PLGA-NPs offered higher 38.4% indel efficacy and 70% PLGA encapsulation efficacy, making them a more effective and secure delivery method for CRISPR components. Copolymer strategy by combination of multiple PNPs could be used as an effective method for CRISPR/Cas system. For instance, PEG-b-PLGA based copolymer with a cationic lipid-assisted NPs achieved direct modulating to immune cells such as neutrophils [164], macrophages [165], and DCs [166], which induced editing efficiency about 25.5–32.7% in vitro. Based on the PEG-b-PLGA combination, PEI-coated PEG-b-PLGA produced 80% selective genome editing in endothelial cells with a 40–50% efficiency [167]. It is evident that the distribution technique of copolymer had a more pronounced editing efficacy than employing PNPs alone. Notably, the helper components, such as cationic lipids or PEI coatings, are crucial to the delivery system that encourages cellular absorption and permits endosomal escape [168]. In-depth research into their interaction in copolymers may be a future direction for PNPs to explore since our understanding of high-efficiency copolymer delivery strategies is still evolving.

Aside from improving editing effectiveness, PNPs may easily implement their multifunctionality because they have effective cargo encapsulation, good modification potential, and interesting stimuli-responsiveness. Due of the exceptional ability of cargo encapsulation, co-delivery strategies that combine medicines and CRISPR components into a single polymer carrier have a beneficial effect in a variety of applications [169, 170]. For instance, the dendrimer-based lipid NPs might co-encapsulate and deliver CRISPR components, including Cas9 mRNA, sgRNA, and donor DNA, as an all-in-one nanocarrier [171]. The findings demonstrated that more than 91% of all cells were altered in vitro with 56% HDR efficiency. In addition, PNPs were frequently used to modify existing delivery vehicles in order to provide them exceptional biocompatibility, stimuli-responsiveness, loading capacity, and other properties. As an illustration, bifunctionalized aminoguanidine-PEGylated periodic mesoporous organosilica NPs successfully delivered RNPs with 40% effectiveness rate for gene editing. In this study, PEG coatings provided the carriers with protection against opsonization, aggregation, and phagocytosis as well as many biofunctions, including exceptional storage stability, permeability, and long-lasting blood circulation of nanocarriers [172, 173]. Numerous stimuli-responsive PNPs delivery systems have been created to extend therapeutic efficacy at a lower dose frequency. These systems can remotely initiate the release of bioactive molecules in response to internal and external stimulation, such as second near-infrared light (NIR II) and pH change [174, 175]. CRISPR/Cas components can be remotely released by NIR-light trigger depending on the specified semiconducting polymer brush. Controllable release of delivery system might be assisted by photothermal conversion, which increased their editing efficiency (around 35%) [176]. Additionally, pH-responsive PNPs provide a novel method of delivering Cas9 RNPs and donor DNA to selected organs. Local administration at various sites can simultaneously provide targeted distribution to desired organs such as intravenous, intratracheal, and intramuscular delivery to the liver, lung, and skeletal muscle in mice, respectively [177]. As a result, the administration method may offer a viable strategy for overcoming the barrier of limited biodistribution at intended areas. Designing delivery systems with a low amount of positive charge or shielding the positive charge reversibly would be a promising avenue to develop a secure and effective platform in the future because most cationic polymeric materials may cause high cytotoxicity.

#### Gold NPs

Inorganic gold nanocarriers have been utilised extensively to deliver imaging agents, nucleic acids, and proteins because of their distinctive physicochemical qualities that are favourable size, minimal toxicity, optical properties, superior biocompatibility, and photothermal action [178, 179]. Among these, the most unique quality that triggers cargo release to control the expression and activity of Cas9 proteins is the photothermal effect provided by AuNPs core [180–182]. Converted heat promoted an endonuclear change of heat-shock factor (HSF) from inactive monomer to active trimer through external NIR laser irradiation. The combination transfection of AuNPs and Cas9 plasmid under the influence of active HSF could achieve 90% GFP-positive cells, which was significantly greater than the result of lipofectamine 2000 [183]. Similarly, after hybridising protective DNA-modified gold nanorod (GNR) with sgRNA, heat damaged the hybrids and released sgRNA into cells under regulated NIR laser irradiation [184]. Furthermore, the unique photothermal activity of AuNPs makes them an appealing candidate for disease treatment, owing to their synergistic photothermal/gene therapy effects. By way of illustration, the mCas9-sGNR nanocarrier not only used to transport CRISPR components needed for gene therapy, but it also lowered the tolerance of cancer cells to heat through photothermal therapy, which led to superadditive synergistic anticancer effects [185]. The development of AuNPs-based CRISPR-Cas12a/Cas13a systems as visual biosensors for large-scale population screening and nucleic acid bioimaging, including smartphone-based diagnostics [186–188]. Compared to earlier detection technology, these devices offer quick, ultrasensitive, specific, and on-site biosensing methods. AuNPs have also been thoroughly studied for tumour visualisation, cancer detection, and bioimaging due to their remarkable photoluminescent characteristics, with the ultimate goal of obtaining precision targeted therapy [189].

## Microalgae-based delivery systems

### AMNPs features

Nanotechnology has grown exponentially as an interdisciplinary field due to its exceptional size and properties. NPs proved to be an excellent material than other counterparts and may serve as the main target in pharmacology, biosensors, and medicine [190, 191]. Synthetic metallic NPs, which exhibit a range of unprecedented physical, chemical, and optoelectronic capabilities, have become effective tools in various sectors over the past ten years. The synthetic procedure is the most important component in evaluating their applicability. Although various cutting-edge techniques were used to boost performance of NPs, such as reliability and practicality, the consensus view is that traditional synthesis of NPs will have a significant negative influence on human well-being because their synthesis was not eco-friendly. Several hazardous reagents, including reducing agents, organic solvents, and stabilisers, are introduced during the synthesis of NPs. These reagents led to significant toxicity issues as well as undesirable byproducts. These synthetic techniques nevertheless have several drawbacks, including high cost, poor efficacy, and requirement of skilled manipulation. The tendency to use sustainable, affordable, and environmental friendly NPs, such as silver, gold, copper, and other metals, would be further increased as a result of the expanding momentum of green chemistry [192]. With regard to biosynthetic mediation, there are the remarkable capabilities to consume, accumulate, and eventually remodel metal ions into NPs, which reduce the toxicity of metal ions and minimise adverse environmental effects through the resistance mechanism by organisms [193]. Biosynthesized NPs have previously been used in antimicrobials, pathogen detection or associated protein identification, and cargo delivery [194].

Bacteria, algae, fungi, and their extracts are emerging as attractive biocatalysts for NPs synthesis. Algae also contain large amounts of natural biocompounds such phytochemicals, carotenoids, vitamins, and pigments. All of which could act as reducing and stabilising substances to hyper-accumulate metal ions and transform them into more flexible forms at the nanoscale [195]. Algae gradually emerged as the best candidate for green synthetic NPs due to their capacity for bioreduction or biosorption. A new term, phyconanotechnology, which combines phycology with nanotechnology, is proposed to describe this phenomenon [196, 197].

Although there has been substantial development in AMNPs, there are still several obstacles existing, such as the strict criteria for homogeneous NP size and shape, reduced kinetics, and difficulties in large-scale manufacture. Managing the NPs development, stability, and aggregation is essential for obtaining uniform NPs and this involves optimisation of the variables, including pH, temperature, concentration, and others [198]. Notably, multidimensional characterizations of generated NPs are a necessary step to evaluate their uses in the interdisciplinary field, as they could provide specific criteria that make NP management easier. Moreover, generating genetic modified strains or screening high-producing algae strains are attractive strategies to synthesis NPs at large scale. Importantly, due to their exceptional biocompatibility in a homologous relationship, AMNPs represent a viable substitute for the administration of medicines, bioactive macromolecules, and gene-editing components, particularly in algae. Thus, future research on the size distribution and aggregation of NPs, as well as their surface properties, morphology, and dissolution rate, will shed light on how to regulate their release for gene editing.

### AMNPs biosynthesis

Top-down and bottom-up approaches are typically used in classical NPs synthesis. The former refers to the transformation of bulk material into thinner crystallites, whilst the later refers to the production of particles by assembling ultra-small building blocks. In terms of effectiveness, tunability, and environmental friendliness, bottom-up technique is best for green synthesis of NPs [199, 200]. Following the natural bio-mineralization process, the major step in synthesising AMNPs is to capture target metal ions from the surrounding environment and enzymatically reduce them to nanoscale. AMNPs can be formed intracellularly or extracellularly, depending on the origins. The intracellular pathway is a very complex and dynamic biological environment in which positively charged metal ions are absorbed by electrostatic interactions to negatively charged cell walls. They were then transformed into metallic NPs by intracellular enzymes [201]. Algae-based metabolic pathways eliminate the need for pre-treatment and give AMNPs high colloidal stability, which benefite from increased biocompatibility and steric stabilisation of bioactive compounds [202, 203]. However, downstream separation of nanomaterials originated from the living cells is still challenging [204].

Extracellular NP synthesis, on the other hand, avoids the additional downstream processing steps and is thus more promising for a variety of applications [205, 206]. Typically, the biosynthesis of NPs comprised two steps: the preparation of algae extracts and metal precursor solution, followed by their incubation for the synthesis reaction [207]. Following incubation, AMNPs have a tendency to aggregate, giving them a thermodynamic stability with different sizes and shapes [207, 208]. The successful synthesis of NPs is indicated by the colour changes of cultured mixture. Despite extracellular synthesis of metallic NPs is easy to harvest in large-scale production, it may grow slower than that of intracellular biosynthesis under the circumstance of cell-free extract. The primary reason may be due to the binding of the active proteins with intracellularly produced NPs rather than extracellularly, which suggests that the active proteins have a special function distinct from cell-free synthesis. As a result, algae have been recognised as the ideal bio-based substrate for the extracellular synthesis of NPs due to their abundance of bioactive components [209]. Despite the fact that NPs have a wide range of uses, efficient, environmental friendly, and easy to scale up, but their synthesis mechanisms are not well understood [210].

### Applications of microalgae-based NPs in delivery

**Fig. 2:**
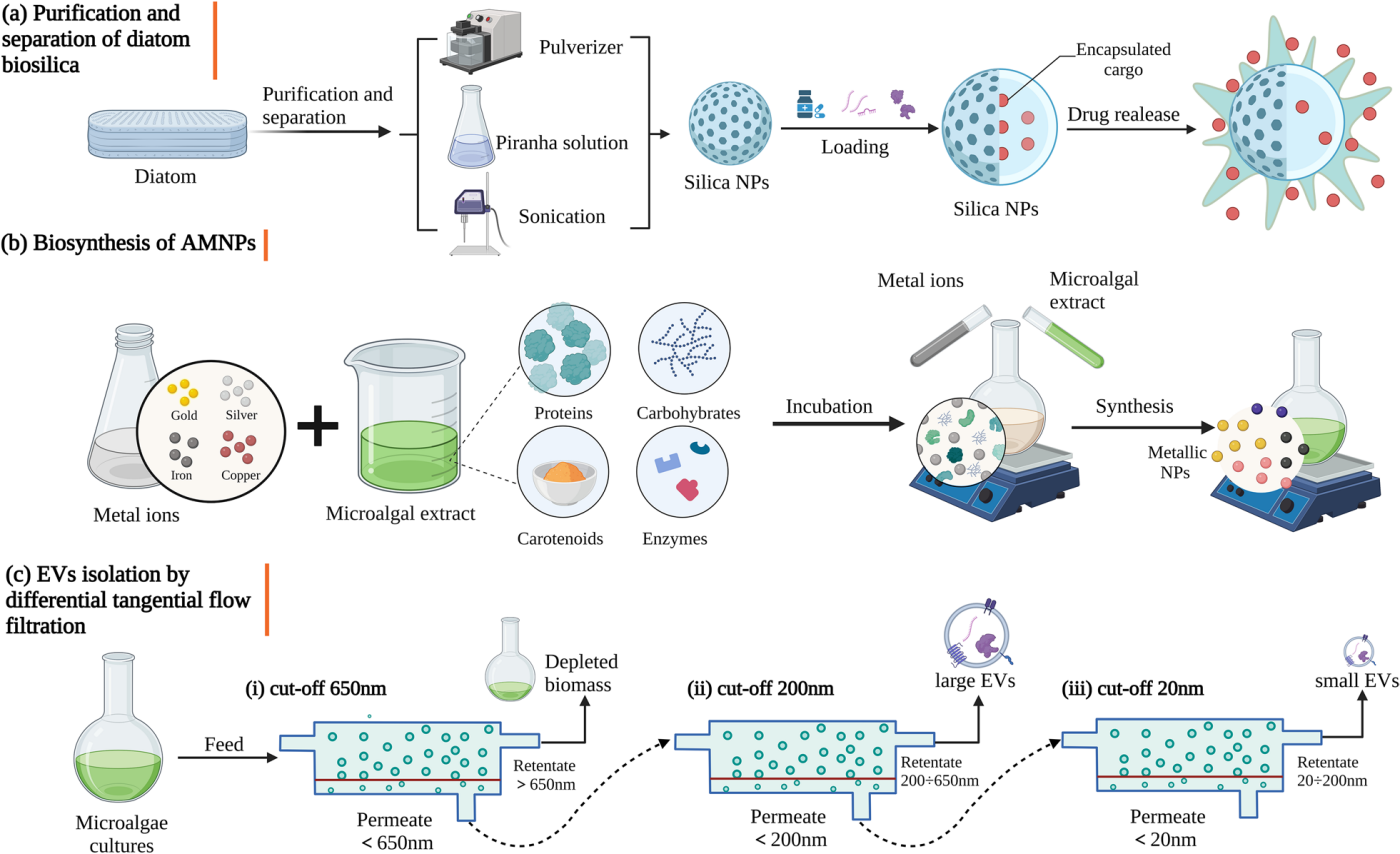
Preparation of microalgae-based nanomaterials. **a** Purification and separation of diatom biosilica. **b** Biosynthesis of AMNPs. **c** EVs isolation by differential tangential flow filtration

The ideal drug delivery systems (DDSs) primarily use nanocarriers with the requirements of safety, effectiveness, and optimal bioavailability to improve the selectivity and targeting of various cargoes into specific cells [211]. However, the shortcomings of conventional NPs, such as their poor stability, high cost, time-consuming, and toxicity, are becoming increasingly apparent in tandem with the increasing demand for nano-DDSs [212, 213]. Many marine bioactive compounds have been employed as reducing and stabilising agents based on the extracellular synthesis of NPs to create metallic nano-DDSs with remarkable biocompatibility, biodegradability, minimal immunogenicity, and non-toxicity [214, 215] (Fig. 2b). Marine carbohydrates have been shown to be the suitable substrates for the construction of AMNPs as DDSs due to considerable advantages in terms of biodiversity, diverse biological activities, and ease of preparation [216, 217]. Bioinspired multifunctional DDSs have been synthesized through extracellular synthesis using the carbohydrates found in marine algae, such as fucoidan [218], resveratrol [219], porphyrin [220], carrageenan oligosaccharide [221], and chitosan [222, 223]. These carbohydrate-based NPs have an exceptional 60%–92% drug loading efficiency, enabling a promising use in the delivery of the CRISPR/Cas system. Nonetheless, the number and type of algae-derived carbohydrates NPs are limited only to a few metals, like AuNPs and AgNPs. Therefore, additional research should be done to determine the other active components from algae that could be used for various types of cargo [15]. In contrast to metallic NPs, marine carbohydrates-based DDSs may be created as carriers through self-assembly or covalent crosslinking and combine the benefits of nanoscale systems with the characteristics of carbohydrates, such as targeted delivery, high biocompatibility, structural modularity, and biodegradability [224].

Aside from that, extracellular vesicles (EVs) generated from microalgae and diatom biosilica are also efficient biological delivery nanomaterials. Diatom-derived biosilica has high specific surface area, excellent drug-holding capacities and biocompatibility, and tailorable surface functionalization for tunable properties because it is encased in a porous 3D nanopatterned silica structure made from biosilica that self-assembled into intricate porous shells [225]. As a result, biosilica can be employed as a sophisticated microcarrier for targeted distribution for therapeutic and medical imaging purposes which is a cost effective and environmental friendly process [226–228]. Biosilica has been created using pulverisation and the strong oxidising capabilities of piranha solution, with the advantages of being cost-effective, easy, quick, and environmental friendly [227] (Fig. 2a). Cargoes could be loaded both on the surface and inside the biosilica NPs, which have a substantial impact on the releasing kinetics of active compounds due to their hierarchical 3D silica porous architectures [229]. More importantly, increased surface modification activities will aid in the targeted delivery of cargoes and the synthesis of multifunctional smart biosilica NPs [230]. For example, chitosan molecules grafted biosilica demonstrated consistent pH responsiveness and notable drug load efficiency with about 90% [231]. EVs, on the other hand, have been widely exploited as the potent tools for cargoes delivery due to their intercellular communication capabilities [232]. Microalgae are an abundant and sustainable source of natural EVs with diverse bioactivities, therefore they are positioned to become competitive competitors in innovative delivery systems [233, 234] (Fig. 2c).

### Microalgae-based biohybrid microrobots

**Fig. 3:**
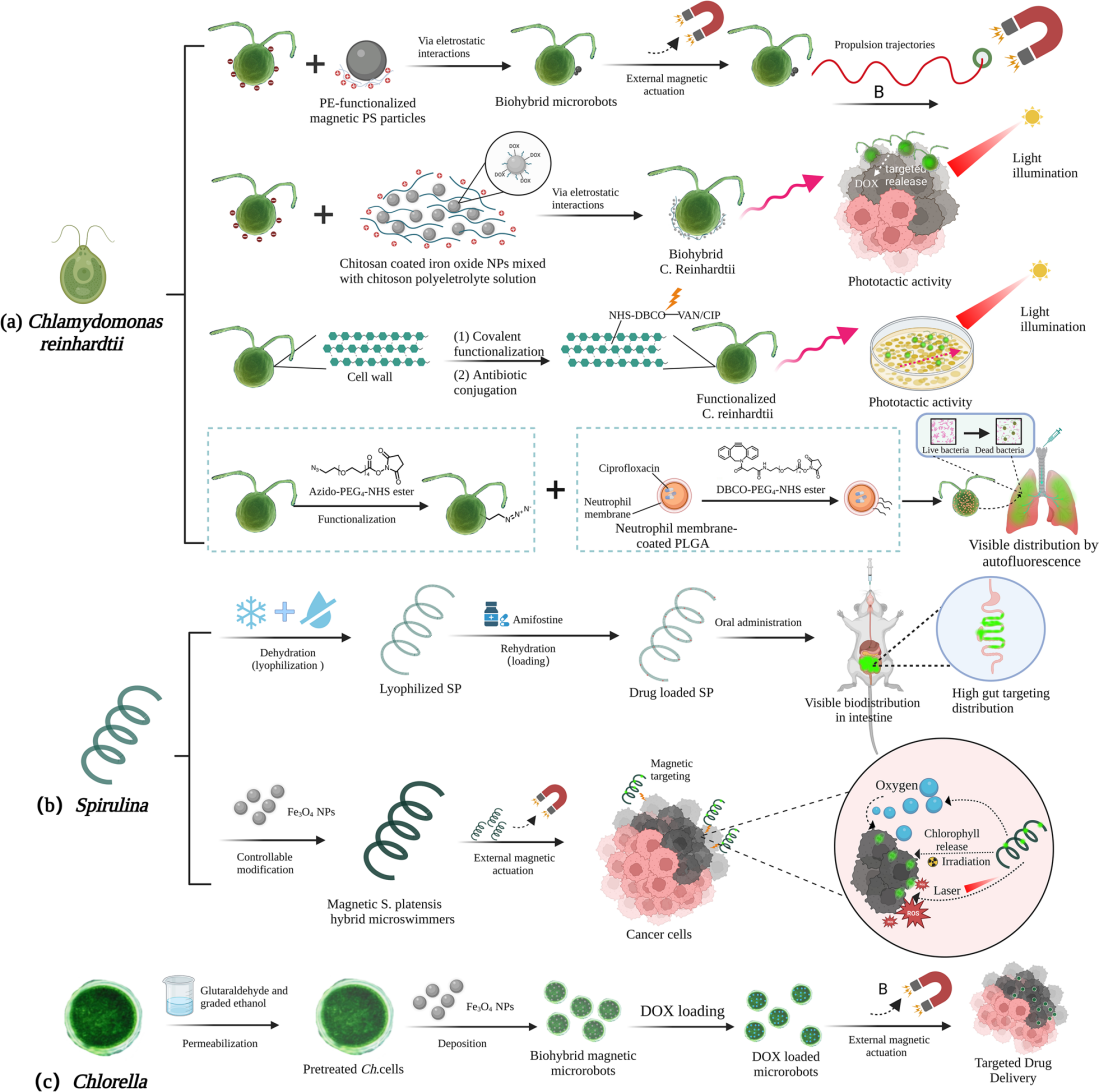
Preparation and application of microalgae-based biohybrid microrobots. **a**–**c** represents the preparation process and application of microalgae-based biohybrid microrobots, including the *Chlamydomonas reinhardtii* (**a)***, Spirulina* (**b**)*,* and *Chlorella* (**c**) etc.

Aside from the previously described microalgae-based nanocarriers, living microalgae-based delivery systems have also exhibited impressive delivery capabilities [235]. Microalgae-based biohybrid microrobots have a number of benefits over conventional synthetic NPs, such as effective and controllable drug delivery, improved stability and bioavailability of therapeutic agents, decreased toxicity and side effects, customizability, eco-friendliness, as well as cost-effectiveness [236, 237]. These microrobots were propelled by magnetic fields or light beams and had high propulsion speeds and phototactic guidance skills, allowing for precise navigation to tissues and difficult-to-reach bodily cavities [238]. As illustrated in Fig. 3, *Chlamydomonas reinhardtii*, *Spirulina*, and *Chlorella* are gaining popularity for use in the construction of microrobots [239, 240]. The modifications and cargo loading of microrobots have negligible influence on their motion, thereby paving the way for the development of living delivery vehicles with enormous potential. Through external magnetically actuated control [241–245], Fe_3_O_4_ magnetized *Chlorella* microrobot demonstrated a greater drug loading efficiency of 98.2% and excellent targeting delivery capabilities (90% target cell death) [246]. The superior drug loading efficiency may be attributed to unique permeation pretreatment and highly negative zeta potential, providing new insights into the preparation of other microalgae-based microrobots.

Additionally, light-driven microalgal microrobots have shown great potential due to their rapid phototaxis response. Numerous tests have shown that biohybrid microrobots are resilient and can be directed towards light stimuli with powerful and precise motion. Although microalgal microrobots can be loaded with drugs with a high manufacturing yield of approximately 90% [247], the drug release effectiveness of microalgal microrobots is inadequate, ranging only from 5 to 10% [248]. Thus, improving drug release efficiency is a critical step towards expanding the use of microalgal microrobots. Microalgal microrobots have also displayed excellent delivery capabilities in local tissues without external targeted actuation. An example is the modification of *Chlamydomonas reinhardtii* with neutrophil membrane-coated and drug-loaded PNPs which allowed antibiotics to achieve about 90% drug release efficiency in the lungs over the first 20 h [235]. Once the issue of low drug release efficiency is resolved, microalgal microrobots will be able to deliver drugs in vivo in an effective, targeted, and safe ways. Furthermore, microalgal microrobots have outstanding fluorescence imaging capabilities that enable non-invasive tracking and real-time monitoring in vivo. They can also overcome hypoxia in tissue engineering and generate cytotoxic reactive oxygen through photodynamic treatment [249]. In summary, the development of microalgal microrobots has opened up new opportunities as a flexible platform for targeted distribution, fluorescence imaging, photothermal therapy, radiotherapy, and other biotechnology applications.

## Current issues and future prospects

### Current drawbacks of gene editing in microalgae

Although CRISPR/Cas systems have demonstrated great promise in the field of microalgae, their application may be hampered by a lack of understanding of microalgal biology, inefficient delivery of genetic materials, decreased expression of Cas9 due to unknown factors, low repair efficiency of the homology-directed repair (HDR) pathway, and other factors [250]. (1) Microalgae bioengineering is still in its infancy, with only a few fully sequenced genome species [251]. In the field of microalgal research, artificial intelligence (AI) has gained popularity and can be used to predict gene sequencing and editing. In this way, artificial intelligence (AI) technology fills the gap between commercial microalgae applications and genetic engineering [252, 253]. (2) It is believed that the cell wall of microalgal cells is the major barrier that reduces the delivery effectiveness of genetic materials during the introduction of macromolecules [254]. In comparison to conventional physical pretreatments like ultrasonication, high-pressure homogenization, bead milling, cryogenic grinding, and pulsed electric field, or mechanical pretreatments like acid, alkaline, and strong oxidant [255], some cutting-edge techniques, such as zinc oxide nanowire array microdevice system and optimised droplet electroporation, are significantly more effective for algal cells with rigid cell walls [256]. It is also necessary to use the right and appropriate delivery vector to increase the effectiveness of genetic materials' transport, and AMNPs have a particularly high potential for microalgae genetic editing with improved biocompatibility [15]. (3). Many approaches have been suggested to combat off-target effect of CRISPR/Cas system, for example reasonable design of sgRNA using different computational tools, like CRISPR-P 2.0 [257], E-CRISP [258], and CasFinder [259], further modification of sgRNA, application of anti-CRISPR proteins as well as appropriate editing conditions (time and temperature). Meanwhile, the cytotoxic effects of Cas9 nuclease could be mitigated by substituting it with Cas12a (4) The expression of Cas9 proteins could be reduced by unknown circumstances. Inserting introns into the Cas9 coding region could improve the efficacy of gene editing, which would significantly improve Cas9 expression [260]. (5) Due to competition from the NHEJ pathway, the HDR pathway is typically less effective in microalgae genome editing for DSBs repair in the host [261]. Given this, various approaches to improving HDR pathway repair efficiency have been suggested, including inhibition of NHEJ pathway [262], regulation of HDR-related factors [263], cell cycle synchronization [264], design of donor DNA template [265], and proximity of CRISPR component and donor DNA template [266].

### Natural ingredient improvement

**Fig. 4:**
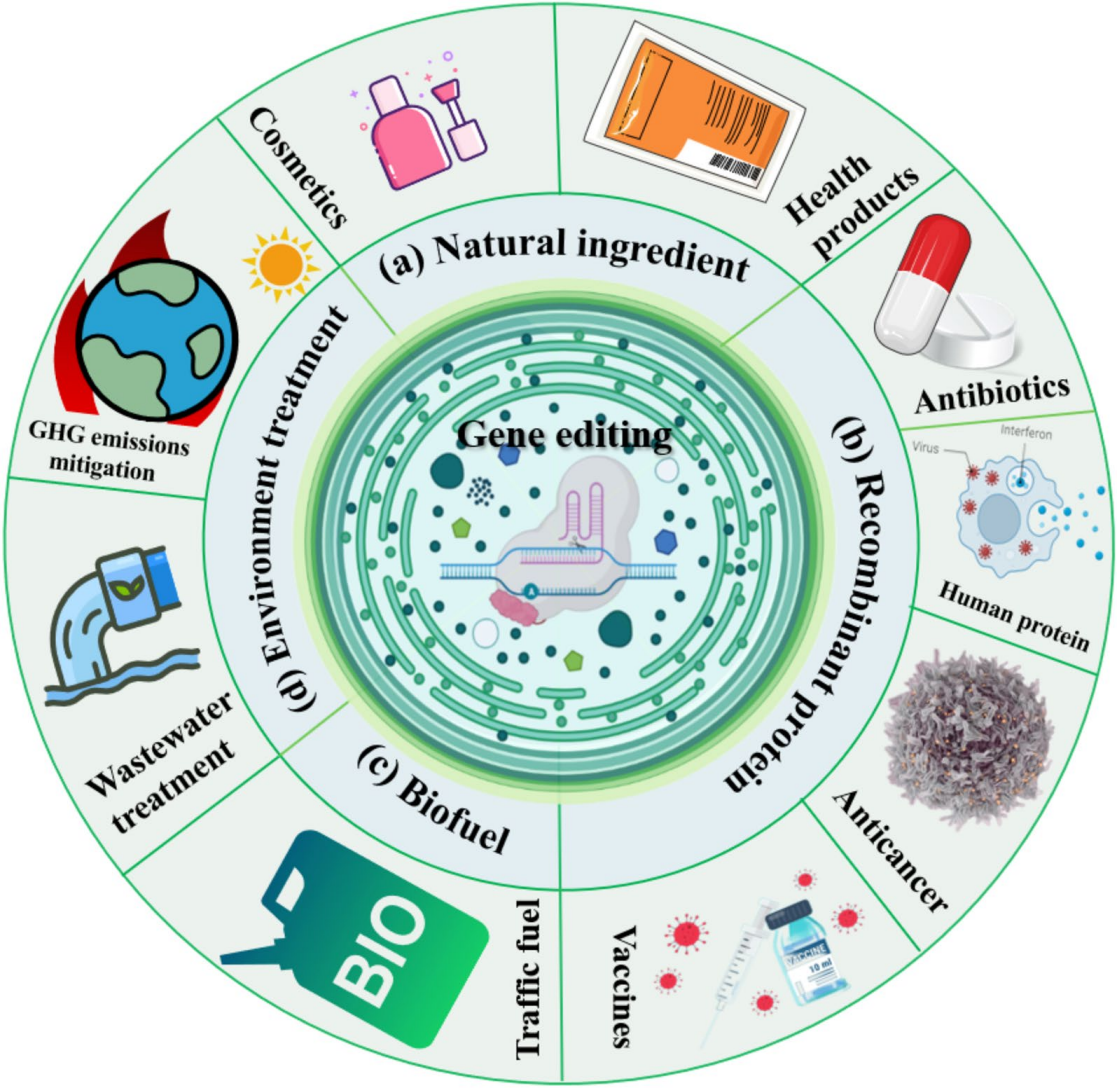
Extensive applications of gene editing microalgae. **a** Enhancing the production of natural ingredient in microalgae. **b** Extending the expression profile of microalgae systems. **c** Exploration of microalgae biofuel. **d** Enhancing the removal efficiency for wastewater and reduction in carbon dioxide emissions

Currently, microalgae are potential sources of valuable bioactive compounds that can be used in different sectors, such as pharmaceutical, cosmetic, and nutraceutical industries [267]. Microalgae could replace higher plants due to its rapid growth, high biomass yield, minimal water use, daily harvest, lack of seasonal restrictions, and ease of culture in in environments with significant climate change [252]. However, most of the biomolecules are produced in relatively small amounts, making it difficult to meet industrial demands [268]. The CRISPR/Cas system is a promising way to boost the production of bioactive molecules with the benefits of pinpoint accuracy, high editing efficiency, multiplex knock-in/out capability, low cost, and short cycle [268, 269] (Fig. 4a). It is ideal to combine genetic and metabolic engineering with omic technologies (transcriptome analysis, proteomics, and liquid chromatography mass spectroscopy) for the purpose of identifying the dynamic molecular regulatory mechanisms and genetic networks involved in the various metabolic pathways in microalgae [256, 270]. Inducing targeted regulation and modification in microalgal cells could be used to precisely control the metabolic pathway and acquire the strains that can produce high-yield targeted compounds that are competitive with present commercial sources.

### Extending expression profile of microalgae systems

Production of recombinant biopharmaceuticals, including vaccines, antibodies, enzymes, hormones, antibiotics, cytokines, thrombolytic agents, and other proteins with medicinal value, is a rapidly growing market for disease prevention and treatment [271] (Fig. 4b). Typically, these proteins are produced by bacteria, yeast, fungus, mammalian cells, and insect, but they have drawbacks, such as expensive, poor yields, prone to contamination, fragile, and trigger immunogenic effects [272]. Eukaryotic microalgae, in contrast, have a shorter culture cycle and are less impacted by seasonal weather conditions in addition to being able to complete complex protein folding and modification to produce active proteins [271]. Both the nucleus and the chloroplast of microalgae can be genetically modified at the same time to produce biopharmaceuticals, providing a large-scale platform for recombinant protein production and industrial use [273].

#### Antibiotics

Antibiotic overuse and regulatory limits are driving an increase in research for antibiotic alternatives, particularly for antibiotic-resistant bacteria. Small oligopeptides known as antimicrobial peptides (AMPs) are present in plants, animals, and bacteria and serve as a general defence against pathogenic microorganisms [274, 275]. In comparison to other systems, the microalgae expression system can manufacture synthetic antibiotics that have the benefit of being flexible while protecting recombinant peptides within their cell walls from digestion [276]. More importantly, microalgae can effectively suppress the growth of bacterial strains through the interaction with their excretion products, such as fatty acids. [277]. Hence, *C. reinhardtii* and *H. pluvialis* have expressed additional antimicrobial and antifungal proteins (ToAMP4 or antimicrobial peptide piscidin-4 gene). Some of them were produced with yields up to 0.32% of total host soluble proteins with more potent antibacterial action against *E. coli*, *S. enteritidis*, *S. aureus*, and *B. subtilis,* despite the fact that expression had no effect on algal growth [278]. The future green synthesis of antibiotics with microalgae is obviously considerably facilitated by the use of gene editing techniques.

#### Human proteins

Recombinant human proteins could be created using the microalgal nucleus as a new expression system for the purpose of treating diseases where these proteins are deficient or where it is necessary to overexpress specific molecules, such as cytokines, growth factors, and human interferon [279, 280]. Aside from that, more health-promoting proteins, like the human growth hormone, growth factors hVEGF-165, hPDGF-B, and hSDF-1, have been successfully produced in the chloroplast system of *C. reinhardtii*, [281–283]. Chloroplast systems have higher levels of recombinant protein production when compared to nucleus systems. Additionally, the chloroplast system has the ability to express multiple transgenes, precise integration of foreign DNA to any locus, and the absence of gene silencing effects [275]. As a result, more algal-derived human proteins will be synthesised in the future, which could be employed as safe and economical biological agents to treat a variety of illness.

#### Vaccines and antigens

There are many benefits of using microalgae as an expression system for manufacturing antigens and antibodies, including their low cost, the capacity to perform post-translational modifications, and elimination of the need for protein extraction and purification. For instance, a direct vaccination against WSSV infection in shrimps could be created using transgenic *Dunaliella salina* that expresses the viral envelope protein VP28 [284]. In fact, the recombinant protein-based vaccinations are not just limited to aquaculture and have the potential to develop into animal feed as well as human food. For instance, biopharmaceuticals synthesize from microalgae can be employed in immunotherapy for diseases like cancer [285] and SARS-COV-2 [286]. A chimeric protein to treat breast cancer was expressed in the microalgae *Schizochytrium* sp*.* by fusing B-cell epitopes from the tumour related antigens, human epidermal growth factor receptor-2, mucin-like glycoprotein 1, Wilms' tumour antigen, and mammaglobin [287]. These epitopes serve as immunotherapy targets and aid in the synthesis of antibodies and CD8^+^/CD4^+^T cell, which kill the malignant cells [288–290]. Berndt et al. [291] use C. *reinhardtii* to produce recombinant SARS-CoV-2 spike RBD proteins that have a comparable affinity to those expressed in mammalian cells. The findings revealed that it is feasible to use algae to produce functional proteins [286]. In short, antigens and antibodies produced by microalgae could serve a wide variety of applications in the future, including the development of diagnostic tools and the treatment of human, plant, and animal diseases.

### Exploration of microalgae biofuel

Microalgae are a viable resource for biofuel generation and the generation of biodiesel using microalgae biomass is 100 times more efficient than that using higher plants as feedstocks [292] (Fig. 4c). Furthermore, microalgae-based biofuels are compatible with existing gasoline engines, eliminating the need for engine modifications [293]. The viability of biofuel as an alternative to traditional fossil fuels will be determined primarily by its economic benefits. Unfortunately, the actual costs of microalgal biofuels are still deemed higher than conventional fossil oils, owing to limited storage lipids and carbohydrates yield in microalgae mass [294]. Numerous initiatives have been launched in the past to increase the production of microalgae biomass and biofuels, including the selection of suitable microalgae species [267], pretreatment of biomass [295] and application of nanotechnology [296]. However, this field still has several constraints that must be addressed with more robust editing tool, such as low carbon fixation efficiency, low lipid accumulation rate, and long cultivation period [297]. The CRISPR/Cas technology presents the best option for resolving the aforementioned problems in microalgae. Microalgae metabolic pathways, for example, can be altered by raising acetyl coenzyme A carboxylase, lowering enzymatic activity of phospholipase A2, or boosting lipid and carbohydrate production. Omic technologies can be used to identify the underlying dynamic molecular regulatory mechanisms and genetic networks involved in the metabolic pathways [298]. Gene-edited microalgal variations might be used in the future to produce more environmentally friendly and clean energy.

### Wastewater treatment

An increasing number of pollutants found in wastewater effluents, such as pharmaceuticals, endocrine disruptors, industrial chemicals, heavy metals, pesticides, etc., are posing threat to human and environmental health [299]. Activated charcoal, flocculation, chemical precipitation, reverse osmosis, ultraviolet disinfection, ultrafiltration, electro-coagulation, and ion exchange are few examples of methods to treat wastewater [300]. However, these methods are not cost-effective since these methods demand a lot of energy and labour and they are unable to eliminate a variety of emerging pollutants in wastewater completely [301]. Microalgal-bioremediation systems have thus been shown to be an effective strategy in wastewater treatment processes to overcome the limitations of such methods. The advantages of microalgal-bioremediation [302] (Fig. 4d) are that it may effectively remove N and P through photosynthetic processes and CO_2_ sequestration. In the meantime, oxygen is released into the water, increasing the amount of dissolved oxygen [303]. Additionally, microalgae increase the pH and dissolved oxygen in the broth as a result of their photosynthetic activities, which reduces pathogen survival [304]. Microalgae have a variety of appealing advantages, but there are still several obstacles existing which are high turbidity, contamination by other microbes, and difficulties in harvesting microalgae biomass. These issues can be resolved using the methods including genomics, computational biology, proteomics, bioinformatics, molecular modelling, molecular dynamics simulation, and a specialised algorithm for pathways prediction [305]. These combined approaches may provide a fast understanding of pollutant binding, degradation, and absorption [306]. Based on this, more robust microalgal strains could be created using gene editing by altering the related genes that are responsible for the biosynthesis of specific enzymes, increasing the adaptability and biosorption ability. This would further improve the efficiency of wastewater treatment and lower operating costs.

### CO_2_ sequestration

Large-scale human activity and excessive greenhouse gas emissions have caused enormous changes in the environment, including the greenhouse effect. These changes have resulted in global warming, climate change, and changes in other environmental aspects [307]. Critical climate change has sparked initiatives worldwide to cut greenhouse gas emissions, especially CO_2_, with the primary goal of keeping rises in global temperature to 2 ℃ [308]. CO_2_ sequestration is critical for managing climate stability and balancing CO_2_ level in the atmosphere [309]. CO_2_ sequestration by microalgae is the most promising approach for mitigating the consequences of greenhouse emissions [310] (Fig. 4d). Microalgae capture CO_2_ and provide various economic and ecological advantages over conventional terrestrial plants, such as rapid growth and sustainability [311], high photosynthetic efficiency, and cooperation with CO_2_ sequestration and wastewater treatment [312]. Additionally, the resulting microalgae biomass can be used to synthesis high-value biomolecules, such as carbohydrates and lipids to produce byproducts for example biofuels and as chemical precursors [313]. A better understanding of the microalgae metabolic pathway with the establishment of ideal genome editing platform has enabled researchers to increase the photosynthetic efficiency of microalgae chassis cells, optimise the microalgae culture conditions, and increase microalgae biomass and yield [314]. Engineered microalgal cell factories based on synthetic biology methods have made it feasible to increase economic efficiency, lessen the greenhouse effect, and protect the environment.

## Conclusion

Target-specific gene editing methods have substantially helped the microalgae industry, enabling improvements in the fields of natural ingredients enhancement, expression profile extension, wastewater treatment, CO_2_ sequestration, and other related fields. Even though this field has made significant advancements, there are still some problems that prevent the widespread application of this cutting-edge technology. These problems include the difficulty of delivering editing components, lack of knowledge about microalgae biology, ambiguous molecular regulatory mechanisms and genetic networks associated with microalgae, and the ineffectiveness of gene editing tools. In this review, a variety of efficient approaches aimed at resolving those problems have been thoroughly discussed. The synthesis and use of novel AMNPs has the potential to increase the effectiveness of gene editing component delivery while minimising damage to microalgae cells. Due to their intrinsic anti-bacterial, anti-cancer, anti-viral, anti-pollution, and bio-remediation capabilities, AMNPs confer additional competitiveness in the gene editing of microalgae in the realms of biomedicine and environmental remediation. Following extensive study on AMNPs, gene editing in microalgae would offer a plethora of opportunities in a variety of industries.

## Data Availability

Not applicable.

## Abbreviations

AMNPs: Algal-mediated nanoparticles
CRISPR/Cas system: Clustered regularly interspaced short palindromic repeats-associated system
ZFN: Zinc finger nuclease
TALEN: Transcription activator-like effector-based nuclease
NPs: Nanoparticles
NP: Nanoparticle
pDNA: Plasmid DNA
RNPs: Ribonucleoproteins
LNPs: Lipid NPs
PNPs: Polymeric nanoparticles
AuNPs: Gold nanoparticles
ApoE: Apolipoprotein E
SORT: Selective organ targeting
PEI: Polyethylenimine
PLGA: Poly (lactic-co-glycolic-acid)
PBAE: Poly (β-amino ester)
PEG: Poly (ethylene–glycol)
NIR: Near-infrared
GFP: Green fluorescent protein
HSF: Heat-shock factor
GNR: Gold nanorod
DDSs: Drug delivery systems
EVs: Extracellular vehicles
PE: Polyelectrolyte
DOX: Doxorubicin
NHS: N-hydroxysuccinimide
DBCO: Dibenzocyclooctyne
HDR: Homology-directed repair
AI: Artificial intelligence
NHEJ: Nonhomologous end joining
AMPs: Antimicrobial peptides
GHGs: Greenhouse gases
